# Supplementary material for: Complexome profiling of the Chlamydomonas psb28 mutant reveals TEF5 as an early PSII assembly factor
Source: Plant Cell. 2025 Mar 17;37(6):koaf055. doi: 10.1093/plcell/koaf055 (PMC12164590; doi:10.1093/plcell/koaf055)
Supplement: koaf055_Supplementary_Data [file koaf055_supplementary_data.zip › psb28_interactive.zip!/plots/intensity/qualityPlots/peptides/_Cre04.g223450.t1.1.html]

### Cre04.g223450.t1.1

Trivial name:   
SeqCoverage [median]: 6.10  
Mass: 61.44 kDa  
  
 

| Sequence | MissedCleavage | Mass [Da] | wtIntensity | muIntensity | Score |
| --- | --- | --- | --- | --- | --- |
| ASVVGPVR | 0 | 783.46 | 0.00 | 978.74 | 86.09 |
| RPGNAAFVADVVTR | 1 | 1471.79 | 388.43 | 6120.32 | 91.44 |
| TGGSAYATLPDYQGK | 0 | 1527.72 | 0.00 | 393.69 | 131.83 |
|  |  |  | 388.4 | 7492.8 |  |
